# Supplementary material for: Quantitative Evaluation of Endogenous Reference Genes for RT-qPCR and ddPCR Gene Expression Under Polyextreme Conditions Using Anaerobic Halophilic Alkalithermophile Natranaerobius thermophilus
Source: Microorganisms. 2025 Jul 23;13(8):1721. doi: 10.3390/microorganisms13081721 (PMC12388727; doi:10.3390/microorganisms13081721)
Supplement: Supplementary file 1 [file microorganisms-13-01721-s001.zip › Table S1.pdf]

Table S1 Protein expressions and primer sequences used in this study

Gene Name	Protein names	Primer sequence (5′−3′)	Product size (bp)
rsmH	Ribosomal RNA small subunit methyltransferase H	F:TTGCCGATATGCCAGTGTAAGG<br>R:CCAATCGCTCCACTGCTCTTAA	133
pdp	Pyrimidine-nucleoside phosphorylase	F:AAAATCTGCCTCAAGCCAAGC<br>R:ATCTTCTTTGGTTGCCCTGC	131
recA	Recombinase A	F:TTCTGTGGCTGCTCTGGTTCC<br>R:CGGCAGTAGTTCGTGACTTCCT	137
accD	Acetyl-coenzyme A carboxylase carboxyl transferase subunits beta/alpha	F:ACTTCAGGTGCTGGATGTGGTA<br>R:CCGCCTTCACCGATGACTAATG	125
sigA	RNA polymerase sigma factor SigA	F:GTGGTGGATGCTATTGGAGAGG<br>R:TCGTCGTCGGGAGATAGAAGG	149
gyrA	DNA gyrase subunit A	F:GCTATGGTACGCATGGCTCAAG<br>R:CGCATGGCAGCAGCACTATC	101
rpoB	DNA-directed RNA polymerase subunit beta	F:GGTTGACGGTAGGACAGGAGAG<br>R:ACCACCTAGAGGCTGCTGAGTA	148
dnaK	Chaperone protein DnaK	F: GGAGATGAACCTTACTCGTGCC<br>R: CTACCTTGTCCACTTCGTCAGC	125
dcuA	Anaerobic c4-dicarboxylate antiporter, Dcu family	F:CAGTTGCTCTGGTAGGGATA<br>R:AATTCAGGACCTTTCTTTCG	130
mnhE	Cation antiporter	F:TAGTAGTAGGCTCTATTGCCAGTATTG<br>R:AAGGTCGGCGATAAGTCCAT	205
nhaC	Na^+/H^+ antiporter NhaC	F:TGTTATGGGTATCAGGTCCG<br>R:TCATTGACCCTAATACATCGG	291
opuAC	Substrate-binding region of ABC-type glycine betaine transport system	F:TGGCATCCGTTACATTCCTA<br>R:GTTGTTCTCCTATCTTGGTCTCA	131
pimT	O-methyltransferase family 3	F:GTATAGAGCTGGAGACGGGTAA<br>R:TTTCTCGCTAATATCACCATAAA	248
proX	Substrate-binding region of ABC-type glycine betaine transport system	F:TGAAAGCGAATTGGGATAC<br>R:GTACTCACCTTGCGTTACTG	127
trkH	Potassium uptake protein, TrkH family	F:GTTGTTCGCTTGGTCAGGTC<br>R:AGCCCATCCCAGTTCTTGT	102
